# Supplementary material for: Cost of Illness in Young Children: A Prospective Birth Cohort Study
Source: Children (Basel). 2021 Feb 24;8(3):173. doi: 10.3390/children8030173 (PMC7996350; doi:10.3390/children8030173)
Supplement: Supplementary file 1 [file children-08-00173-s001.pdf]

Supplementary figures and tables

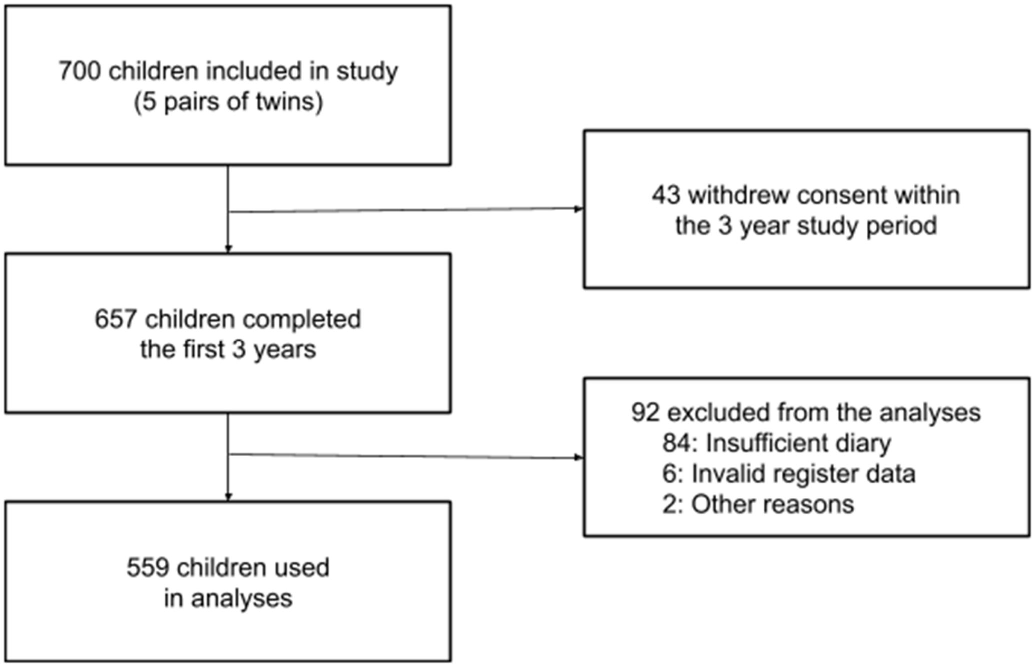

Figure S1. Flow chart. Overview of the selection of the study population.

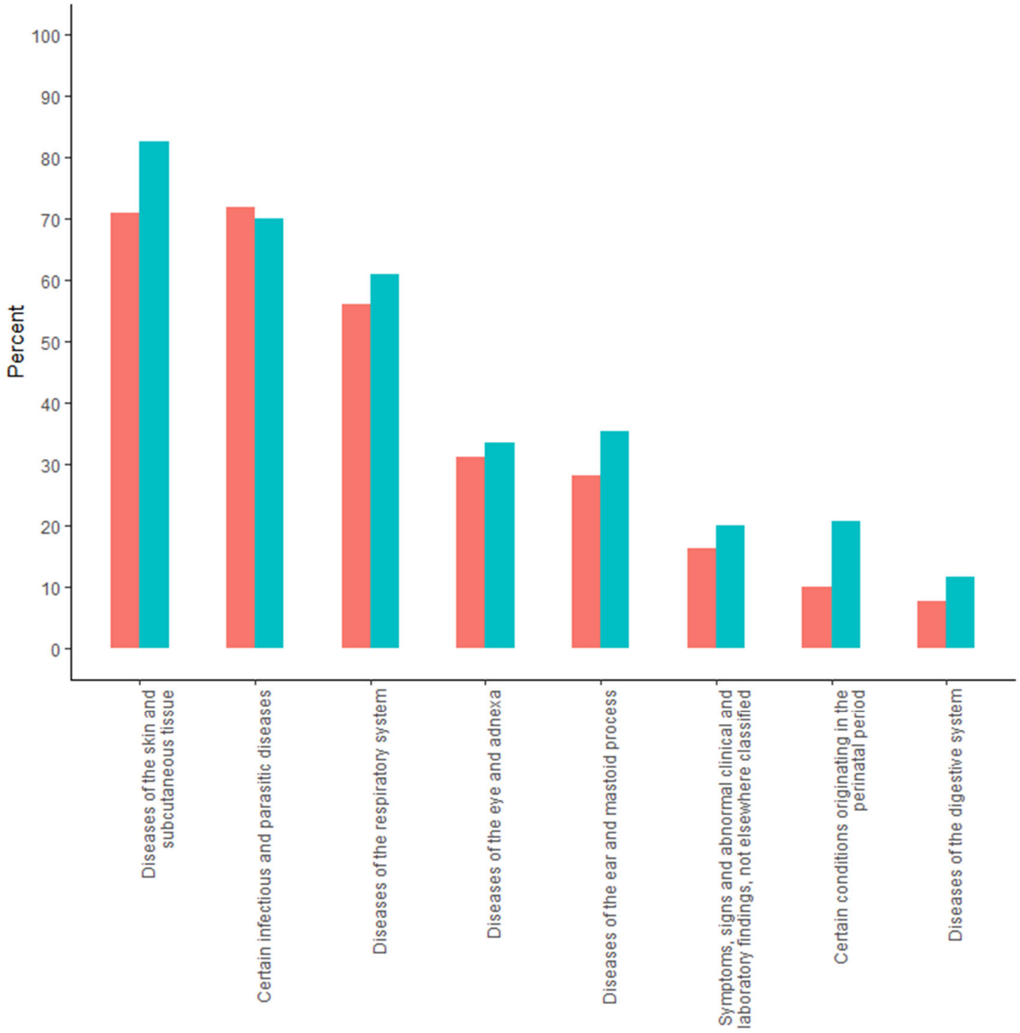

Figure S2. Incidence of disease per 100 children by mode of delivery. Incidence of the eight most common diseases by ICD10 group stratified by mode of delivery. Red: vaginal delivery, green: cesarean delivery

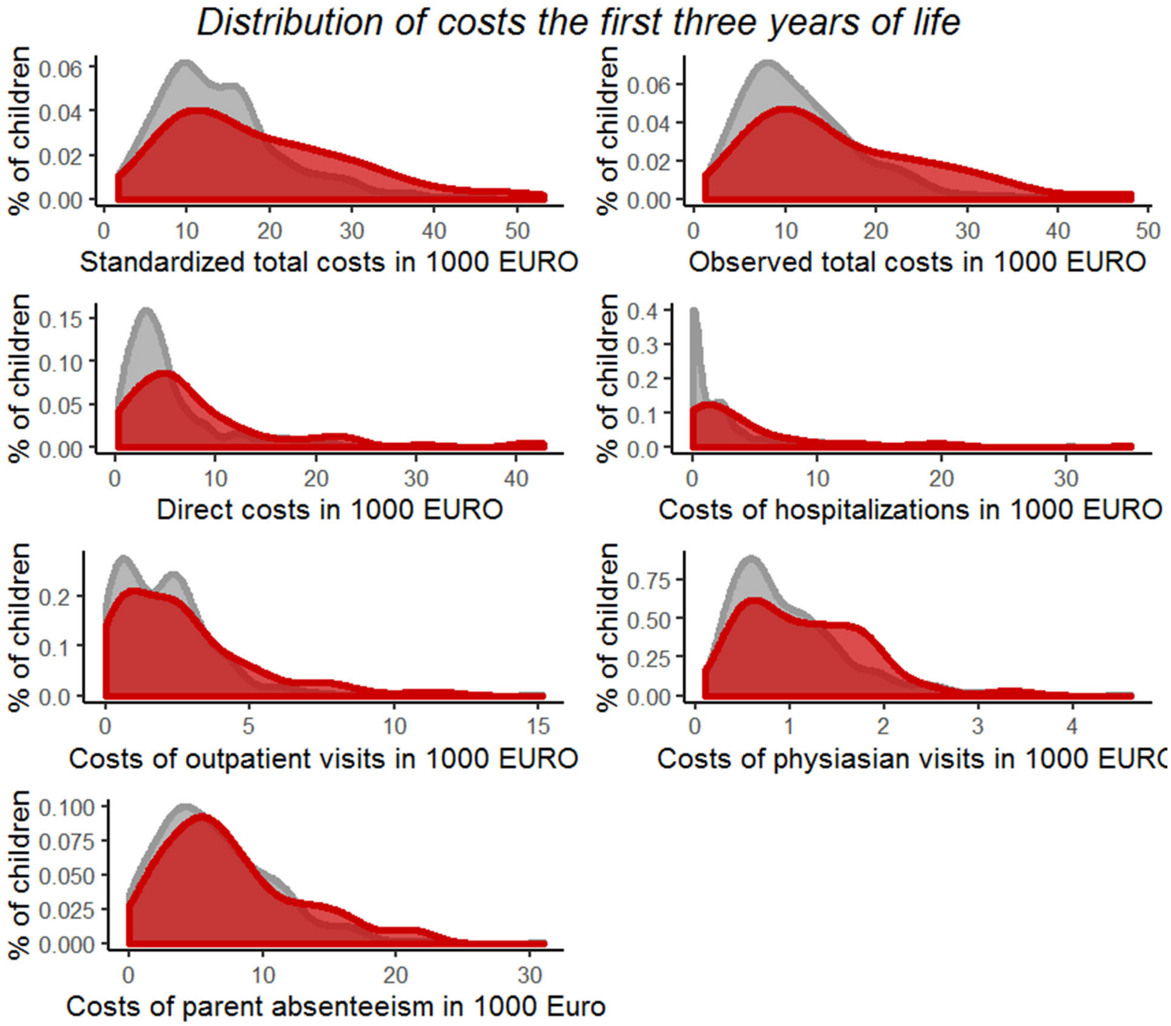

Figure S3. Distributions of costs by mode of delivery. Distributions of costs during the first three years of life stratified by mode of delivery. Red: cesarean delivery, grey: vaginal delivery.

Table S1A Drop-out analysis. A comparison between the study population and the excluded participants.

	Study population (n = 559)	Excluded (n = 141)	P-value*
**Fish-oil supplement, *n* (%)**	282 (50·5)	65 (46·4)	0·44
**High-dose vitamin D supplement, *n* (%)**	230 (41·1)	68 (48·2)	0·15
**Maternal smoking, *n* (%)**	16 (2·9)	9 (6·4)	0·08
**Maternal age (years), mean ± sd**	32·30 ± 4·12	32·21 ± 5·23	0·83
**Maternal atopic disease, *n* (%)**	312 (55·9)	67 (47·5)	0·09
**Maternal pre pregnancy BMI, mean ± sd**	24·56 ± 4·43	24·50 ± 4·26	0·89
**Preeclampsia, *n* (%)**	29 (5·2)	3 (2·1)	0·18
**Caesarean section, *n* (%)**	110 (19·7)	41 (29·1)	**0·021**
**Male, *n* (%)**	282 (50·4)	78 (55·3)	0·35
**Premature, *n* (%)**	21 (3·8)	7 (5·0)	0·68
**GA (weeks), mean ± sd**	39·87 ± 1·71	39·82 ± 1·47	0·79
**Birth weight (100g), mean ± sd**	35·26 ± 5·52	35·75 ± 5·38	0·35
**Season of birth, *n* (%)**			0·56
**Summer**	114 (20·4)	35 (24·8)	
**Autumn**	120 (21·5)	30 (21·3)	
**Winter**	171 (30·6)	44 (31·2)	
**Spring**	154 (27·5)	32 (22·7)	
**Older siblings, *n* (%)**	313 (56·0)	82 (58·2)	0·71
**Cat at birth, *n* (%)**	111 (19·9)	31 (22·0)	0·66
**Dog at birth, *n* (%)**	97 (17·4)	35 (24·8)	0·06
**Breastfed (months), mean ± sd**	3·42 ± 1·93	3·23 ± 2·05	0·33
**Social circumstances, mean ± sd**	0·02 ± 0·95	-0·08 ± 1·17	0·28
**Day care (years), mean ± sd**	0·90 ± 0·24	0·92 ± 0·32	0·29
**Type of daycare (nursery), *n* (%)**	352 (66·0)	76 (63·3)	0·65

* *p*-value corresponds to chi-squared test for categorical variables and student's t-test continuous variables.

Table S1B Differences between the intervention groups. A comparison between the intervention groups.

Variable	Both	Only fish-oil	Only Vitamin D	Nothing	p-value*
**n**	112	170	118	158	
**Maternal smoking, *n* (%)**	1 (0·9)	8 (4·7)	3 (2·5)	4 (2·5)	0·291
**Maternal age, mean ± sd**	32·17 ± 4·02	32·22 ± 3·90	32·92 ± 4·11	32·03 ± 4·42	0·319
**Maternal atopic disease, *n* (%)**	65 (58·0)	91 (53·8)	70 (59·3)	85 (53·8)	0·720
**Maternal pre pregnancy BMI, mean ± sd**	25·10 ± 4·61	24·61 ± 3·99	24·05 ± 4·79	24·51 ± 4·46	0·356
**Preeclampsia, *n* (%)**	8 (7·1)	7 (4·1)	7 (5·9)	7 (4·5)	0·668
**Cesarean delivery, *n* (%)**	21 (18·8)	36 (21·2)	26 (22·0)	27 (17·1)	0·709
**Male, *n* (%)**	52 (46·4)	82 (48·2)	69 (58·5)	78 (49·4)	0·244
**GA (weeks), mean ± sd**	40·08 ± 1·61	39·99 ± 1·61	39·74 ± 1·95	39·66 ± 1·67	0·127
**Premature, *n* (%)**	4 (3·6)	5 (2·9)	5 (4·2)	7 (4·4)	0·897
**Birth weight (100g), mean ± sd**	35·76 ± 5·35	35·74 ± 5·61	35·19 ± 6·16	34·43 ± 4·96	0·121
**Season of birth (ref: summer), *n* (%)**					**0·003**
**Autumn**	21 (18·8)	38 (22·4)	24 (20·3)	31 (19·6)	
**Winter**	32 (28·6)	33 (19·4)	27 (22·9)	27 (17·1)	
**Spring**	43 (38·4)	39 (22·9)	41 (34·7)	48 (30·4)	
**Summer**	16 (14·3)	60 (35·3)	26 (22·0)	52 (32·9)	
**Older siblings at birth, *n* (%)**	64 (57·1)	101 (59·4)	75 (63·6)	72 (45·6)	**0·014**
**Cat at birth, *n* (%)**	22 (19·6)	36 (21·2)	21 (17·8)	32 (20·3)	0·915
**Dog at birth, *n* (%)**	16 (14·3)	35 (20·6)	15 (12·7)	30 (19·0)	0·255
**Social circumstances, mean ± sd**	0·14 ± 1·00	-0·03 ± 0·87	0·15 ± 0·92	-0·10 ± 1·01	0·075
**Solely breastfed (mths), mean ± sd**	3·32 ± 1·83	3·44 ± 1·97	3·47 ± 1·87	3·42 ± 2·02	0·940
**Introduction to daycare (mht), mean ± sd**	0·87 ± 0·18	0·91 ± 0·28	0·88 ± 0·18	0·92 ± 0·26	0·247
**Type of daycare (nursery), *n* (%)**	72 (67·3)	104 (62·7)	81 (71·7)	94 (64·4)	0·440

**p*-value corresponds to chi-squared test for categorical variables and student's t-test continuous variables.

Table 2. Description of costs. Descriptive statistics of the costs in the study population and the subgroup where the mothers did not receive fish-oil supplementation in pregnancy, stated in 2017-Euro.

		**All**	**Subgroup**
**Cost**	**Year**	**Median (IQR)**	**Median (IQR)**
**Total costs (standardized)**	**Year 1-3**	**13 290 (9 038–18 576)**	**14 061 (9 751–19 662)**
	Year 1	3 467 (2 010-5 640)	3 713 (2 082-6 085)
	Year 2	5 323 (2 979-8 689)	5 538 (3 253-9 201)
	Year 3	3 264 (1 748-5 189)	3 560 (1 875-5 419)
**Total costs (observed)**	**Year 1-3**	**10 932 (7 310-16 276)**	**11 987 (7 861-16 844)**
	Year 1	3 224 (1 875-5 321)	3 312 (2 031-5 488)
	Year 2	4 306 (2 276-7 192)	4 396 (2 380-7 777)
	Year 3	2 496 (1 439-4 221)	2 727 (1 575-4 467)
**Direct Costs**	**Year 1-3**	**4 225 (2 549-7 130)**	**4 372 (2 624-8 265)**
	Year 1	2 488 (886-4 337)	2 538 (1 043-4 655)
	Year 2	819 (362-1 707)	882 (410-1 736)
	Year 3	460 (171-1 035)	556 (226-1 131
**Physician**	**Year 1-3**	**868 (561-1 357)**	**891 (586-1 411)**
	Year 1	323 (211-490)	320 (209-474)
	Year 2	307 (173-550)	316 (184-604)
	Year 3	153 (82-279)	180 (93-305)
**Hospital**	**Year 1-3**	**1 248 (0-3 401)**	**1 255 (0-3 799**
	Year 1	0 (0-2 408)	0 (0-2 470)
	Year 2	0 (0-0)	0 (0-0)
	Year 3	0 (0-0)	0 (0-0)
**Outpatient**	**Year 1-3**	**1 925 (744-2 916)**	**2 126 (883-2 943)**
	Year 1	753 (136-2 058)	863 (103-2 116)
	Year 2	274 (0-777)	383 (0-762)
	Year 3	200 (0-549)	220 (0-663)
**Observed parent absenteeism**	**Year 1-3**	**5 909 (3 399-9 196)**	**6 191 (3 546-9 869)**
	Year 1	156 (0-1 261	214 (0-1 314)
	Year 2	3 039 (1 536-5 403)	3 073 (1 629-5 909)
	Year 3	1 824 (946-3 073)	1 891 (946-3 349)
**Days**	**Year**	**Median (IQR)**	**Median (IQR)**

**Parent absenteeism**	**Year 1-3**	**24 (15-37)**	**26 (16-39·25)**
	Year 1	1 (0-5)	1 (0-5)
	Year 2	13 (7-22)	13 (7-23)
	Year 3	8 (4-13)	8 (4-13·25)

Table S3. Approximated costs of parental absenteeism in different countries. Estimated costs of the environmental factors effect (from table 2) on parental absenteeism in Denmark, England and USA.

	Model estimates	Interpreta-tion	Denmark (2017-Euro)	England (2017-£)	USA (2017-$)
**Variable**	**aOR [95% CI] p-value**	**Days based on GM***			
**High-dose vitamin D supplement**	0·99 [0·88; 1·12] 0·90	-0·2 [-3·0; 3·0]	-65 [-994; 984]	-22 [-333; 329]	-30 [-450; 445]
**Maternal smoking**	1·10 [0·79; 1·57] 0·60	2·5 [-5·5; 14·8]	821 [-1790;4858]	275 [-600;1627]	371 [-810; 2198]
**Maternal age**	1·00 [0·98; 1·01] 0·78	-0·1 [-0·4; 0·3]	-17 [-137; 104]	-6 [-46; 35]	-8 [-62; 47]
**Maternal atopic disease**	1·05 [0·93; 1·18] 0·42	1·3 [-1·7; 4·6]	412 [-566; 1506]	138 [-190; 504]	186 [-256; 681]
**Maternal pre pregnancy BMI**	1·00 [0·99; 1·01] 0·87	0·0 [-0·4; 0·3]	-9 [-118; 104]	-3 [-39; 35]	-4 [-53; 47]
**Preeclampsia**	1·29 [1·01; 1·68] 0·05	7·5 [0·1; 17·6]	2451 [44; 5775]	821 [15; 1934]	1109 [20; 2613]
**Cesarean delivery**	1·17 [1·02; 1·35] **0·0289**	4·5 [0·5; 9·2]	1473 [156; 3031]	493 [52; 1015]	667 [70; 1372]
**Male**	1·03 [0·92; 1·16] 0·58	0·9 [-2·0; 4·1]	284 [-673; 1356]	95 [-225; 454]	128 [-304; 614]
**GA (weeks)**	1·03 [0·99; 1·06] 0·14	0·7 [-0·2; 1·6]	222 [-81; 529]	74 [-27; 177]	101 [-37; 239]
**Premature**	0·86 [0·64; 1·17] 0·31	-3·7 [-9·4; 4·5]	-1229 [-3077; 1474]	-412 [-1030; 494]	-556 [-1392; 667]
**Birth weight (100g)**	1·00	-0·1 [-0·3; 0·2]	-18 [-111; 75]	-6 [-37; 25]	-8 [-50; 34]

	[0·99; 1·01] 0·69				
**Season of birth (ref: summer)**					
**Autumn**	1·05 [0·88; 1·26] 0·56	1·4 [-3·0; 6·7]	459 [-1000; 2199]	154 [-335; 736]	208 [-453; 995]
**Winter**	0·94 [0·80; 1·11] 0·50	-1·4 [-5·2; 2·9]	-472 [-1695; 956]	-158 [-567; 320]	-213 [-767; 432]
**Spring**	1·00 [0·85; 1·18] 0·99	0·0 [-4·0; 4·8]	14 [-1306; 1563]	5 [-437; 523]	6 [-591; 707]
**Older siblings at birth**	1·22 [1·04; 1·44] **0·0146**	1·6 [-1·4; 5·0]	531 [-458; 1637]	178 [-153; 548]	240 [-207; 741]
**Cat at birth**	1·02 [0·83; 1·26] 0·83	0·4 [-3·1; 4·5]	124 [-1020; 1478]	42 [-342; 495]	56 [-462; 669]
**Dog at birth**	0·82 [0·66; 1·03] 0·08	-4·2 [-7·2; -0·5]	-1373 [-2370; -179]	-460 [-794; -60]	-621 [-1072; -81]
**Social circumstances**	1·00 [0·92; 1·09] 0·97	-0·1 [-1·6; 1·5]	-28 [-532; 506]	-9 [-178; 169]	-13 [-241; 229]
**Solely breastfed (mths)**	1·00 [0·96; 1·04] 0·97	0·3 [-0·5; 1·0]	87 [-163; 344]	29 [-55; 115]	40 [-74; 156]
**Introduction to daycare (mht)**	0·96 [0·93; 1·00] **0·0087**	-1·3 [-1·9; -0·7]	-426 [-618; -225]	-143 [-207; -76]	-193 [-280; -102]
**Type of daycare (nursery)**	1·33 [1·12; 1·58] **0·0011**	9 [5·1; 13·3]	2947 [1668; 4371]	987 [559; 1464]	1333 [755; 1978]

Table S4. Associations between environmental factors and costs in placebo strata. Associations between environmental factors, and costs and days absent from daycare the first three years of life in the subpopulation whose mothers did not receive fish-oil supplementation.

	**Standardized total costs**		**Observed total costs**		**Direct costs**		**Days home from daycare**	
Environmental factor	GMR [95% CI] p-value	Interpreta-tion Euro	GMR [95% CI] p-value	Inter-preta-tion Euro	GMR [95% CI] p-value	Inter-preta-tion Euro	OR [95% CI] p-value	Inter-pre-ta-tion Euro
High-dose vita-min D supple-ment	1·10 [0·95; 1·27] 0·19	1380 [-624; 3697]	1·11 [0·95; 1·29] 0·18	1132 [-473; 2994]	1·12 [0·90; 1·39] 0·32	480 [-411; 1586]	0·98 [0·83; 1·16] 0·85	-0·4 [-4·3; 4·2]
Maternal smok-ing	1·27 [0·81;2·01] 0·30	3681 [-2608;13616]	1·12 [0·70; 1·79] 0·64	1257 [-3139; 8294]	1·48 [0·75; 2·94] 0·26	1978 [-1021; 7913]	1·26 [0·78; 2·2] 0·38	6·7 [-5·8; 31·1]
Maternal age	1·00 [0·98; 1·02] 0·99	0 [-224; 227]	1·01 [0·99; 1·03] 0·17	126 [-54; 310]	1·00 [0·98; 1·03] 0·81	13 [-89; 117]	1·00 [0·98; 1·02] 0·74	-0·1 [-0·6; 0·4]
Maternal atopic disease	1·19 [1·03; 1·37] 0·0208	2502 [365; 4969]	1·14 [0·98; 1·32] 0·08	1471 [-173; 3378]	1·36 [1·10; 1·69] 0·0049	1482 [411; 2808]	1·08 [0·91; 1·27] 0·38	2 [-2·3; 7]
Maternal pre pregnancy BMI	1·00 [0·99; 1·02] 0·88	17 [-194; 231]	1·00 [0·98; 1·02] 0·93	-8 [-175; 163]	1·01 [0·99; 1·03] 0·39	42 [-54; 140]	1·00 [0·98; 1·02] 0·81	-0·1 [-0·5; 0·4]
Preeclampsia	1·13 [0·82; 1·57] 0·46	1787 [-2481; 7716]	1·10 [0·79; 1·55] 0·56	1095 [-2215; 5734]	1·07 [0·66; 1·75] 0·78	300 [-1402; 3080]	1·16 [0·81; 1·72] 0·43	4·2 [-4·9; 18·6]
Cesarean deliv-ery	1·30 [1·08; 1·55] 0·0050	4001 [1122; 7449]	1·34 [1·12; 1·61] 0·0020	3577 [1211; 6422]	1·46 [1·11; 1·91] 0·0064	1874 [467; 3716]	1·15 [0·94; 1·42] 0·19	3·9 [-1·6; 10·9]
Male	1·23 [1·07; 1·42] 0·0047	3090 [896; 5620]	1·28 [1·11; 1·48] 0·0011	2908 [1099; 5000]	1·29 [1·05; 1·60] 0·0184	1203 [187; 2461]	1·05 [0·89; 1·23] 0·59	1·2 [-2·9; 6]

GA (weeks)	0·91 [0·88; 0·95] <0·0001	-1148 [-1616; -661]	0·91 [0·87; 0·94] <0·0001	-980 [-1348; -597]	0·81 [0·77; 0·86] <0·0001	-761 [-939; -574]	1·03 [0·98; 1·08] 0·23	0·7 [-0·5; 2]
Premature	2·09 [1·48; 2·94] <0·0001	14662 [6512; 26139]	2·36 [1·67; 3·35] <0·0001	14241 [6978; 24530]	4·62 [2·82; 7·58] <0·0001	14787 [7415; 26885]	0·77 [0·52; 1·17] 0·19	-6·1 [-12·5; 4·5]
Birth weight (100g)	0·98 [0·97; 0·99] 0·0048	-250 [-419; -78]	0·98 [0·97; 0·99] 0·0020	-218 [-352; -82]	0·95 [0·93; 0·97] <0·0001	-194 [-267; -121]	1·01 [0·99; 1·02] 0·39	0·2 [-0·2; 0·6]
Season of birth (ref: summer)								
Autumn	1·34 [1·07; 1·69] 0·011	4636 [965; 9240]	1·37 [1·08; 1·73] 0·0088	3851 [879; 7602]	1·46 [1·04; 2·05] 0·0314	1862 [147; 4272]	1·19 [0·92;1·54] 0·19	5 [-2·1; 14·2]
Winter	1·05 [0·86; 1·29] 0·64	674 [-1922; 3855]	1·06 [0·86; 1·31] 0·56	668 [-1425; 3247]	1·08 [0·80; 1·47] 0·61	336 [-825; 1910]	1·02 [0·81;1·29] 0·85	0·6 [-5; 7·5]
Spring	1·06 [0·86; 1·31] 0·57	841 [-1849; 4153]	1·08 [0·87; 1·33] 0·50	796 [-1372; 3481]	1·13 [0·82; 1·54] 0·46	513 [-722; 2201]	1·11 [0·87;1·41] 0·38	2·9 [-3·3; 10·7]
Older siblings at birth	1·12 [0·97; 1·30] 0·12	1640 [-381; 3973]	1·13 [0·97; 1·31] 0·12	1321 [-296; 3194]	0·95 [0·76; 1·18] 0·62	-214 [-964; 717]	1·22 [1·04; 1·44] 0·0146	5·8 [1·1; 11·5]
Cat at birth	0·98 [0·82; 1·18] 0·84	-245 [-2453; 2405]	0·96 [0·8; 1·16] 0·70	-385 [-2109; 1696]	1·08 [0·82; 1·41] 0·60	310 [-739; 1690]	1·02 [0·83; 1·26] 0·83	0·6 [-4·3; 6·9]
Dog at birth	0·97 [0·8; 1·17] 0·73	-455 [-2757; 2344]	0·94 [0·77; 1·15] 0·56	-599 [-2387; 1586]	1·33 [1·00; 1·78] 0·05	1346 [-18; 3168]	0·82 [0·66; 1·03] 0·08	-4·6 [-8·8; 0·8]
Social circum-stances	0·98 [0·91; 1·06] 0·67	-215 [-1156; 797]	1·08 [1·01; 1·17] 0·0359	880 [60; 1764]	0·92 [0·83; 1·03] 0·16	-310 [-702; 127]	1·00 [0·92; 1·09] 0·97	0 [-2·1; 2·2]

Solely breastfed (mths)	0·99 [0·96; 1·03] 0·74	-83 [-569; 420]	0·99 [0·96; 1·03] 0·70	-77 [-464; 325]	0·98 [0·92; 1·03] 0·38	-101 [-315; 125]	1·00 [0·96; 1·04] 0·97	0 [-1·1; 1·1]
Introduction to daycare (mht)	0·98 [0·95; 1·00] 0·09	-300 [-640; 48]	0·98 [0·96; 1·01] 0·19	-187 [-459; 93]	1·00 [0·96; 1·04] 0·90	10 [-148; 174]	0·96 [0·93; 1·00] 0·0087	-1 [-1·9; -0·1]
Type of daycare (nursery)	1·22 [1·04; 1·43] 0·0150	2933 [540; 5736]	1·25 [1·06; 1·47] 0·0080	2587 [636; 4881]	0·84 [0·66; 1·07] 0·15	-651 [-1379; 271]	1·33 [1·12; 1·58] 0·0011	8·6 [3·1; 15]

Table S5. Baseline table of original information used to derive the measure of social circumstances. Baseline distribution of the original information used to derive the measure of social circumstances of the 700 children included in the $COPSAC_{2010}$ cohort.

	**$COPSAC_{2010}$** ***n* = 700**
**Household income**	***n* (%)**
Below 400 000	60 (9)
400 000 – 600 000	131 (19)
600 000 – 800 000	245 (35)
800 000 – 1 000 000	136 (19)
Above 1 000 000	128 (18)
**Maternal education**	***n* (%)**
Elementary or College	51 (7)
Medium or Tradesman	444 (63)
University	205 (29)
**Maternal age, mean ± sd**	34.28 ± 4.36
